# The Analysis of E-Cadherin, N-Cadherin, Vimentin, HER-2, CEA, CA15-3 and SF Expression in the Diagnosis of Canine Mammary Tumors

**DOI:** 10.3390/ani12213050

**Published:** 2022-11-06

**Authors:** Chao Yu, Huihua Zheng, Xiangyu Liu, Guanghong Xie

**Affiliations:** College of Veterinary Medicine, Jilin University, No. 5333 Xi’an Road, Changchun 130062, China

**Keywords:** canine mammary tumors, molecular biomarkers, histopathology, imagiology

## Abstract

**Simple Summary:**

Canine mammary tumors (CMTs) are the most common neoplasms in female dogs, and their high rate of recurrence and metastasis result in a poor prognosis; therefore, timely and effective diagnosis is essential. In this study, the tumors were identified by histopathology and combined with X-ray and ultrasonography to assess the presence of organ metastases. Changes in certain indicators that suggested multiple direct or paraneoplastic changes associated with tumors were detected/suspected by hematological examination. Enzyme-linked immunosorbent assay (ELISA) revealed that HER-2 serum concentrations were significantly different between healthy dogs and dogs with malignant tumors. mRNA expression of HER-2, E-cadherin, N-cadherin, Vimentin, CEA, CA15-3 and SF were measured in CMT tissues by qPCR. The expression of these tumor markers (except for E-cadherin) in the malignant tumor group was significantly higher than in the benign group and the healthy control group (*p* < 0.05), however, there were no significant differences between the benign mammary tumor group and the healthy control group (*p* > 0.05). Measurement of biomarkers in dogs by serological and molecular biological assays represents a milestone in the early diagnosis of tumors, assessment of disease progression and response to chemotherapy.

**Abstract:**

Canine mammary tumors (CMTs) are one of the most common tumors in female dogs, and they are associated with a poor prognosis owing to their high rate of recurrence and metastasis rates, which make their diagnosis especially important in clinical veterinary medicine. In this study, the characteristics of tumors were observed in dogs suffering from CMTs, and clinical diagnosis and histopathology were used to identify tumors. Furthermore, the expression levels of tumor markers for CMTs were analyzed by enzyme-linked immunosorbent assay (ELISA) and quantitative PCR (qPCR). Upon clinical examination, dogs with CMTs displayed a distinct and irregular mass in the mammary gland, and X-ray (Latero-lateral and ventro-dorsal views) and ultrasonography of the abdomen revealed a moderately echogenic mass at the mammary gland with slightly stronger density than the surrounding tissue. A total of 30 tumors were identified by histopathology, 11 benign and 19 malignant. Changes in some indicators in dogs suffering from CMTs and healthy dogs suggested that there were multiple direct or paraneoplastic changes associated with tumors that could be detected/suspected by hematological examination, and ELISA revealed the HER-2 serum concentrations were significantly different between healthy animals and those with malignant tumors. qPCR indicated that tumor markers N-cadherin, Vimentin, HER-2, CEA, CA15-3 and SF were higher in dogs with malignant tumors than healthy dogs, with a low level of E-cadherin in malignant tumors. This study verified that serological tests and molecular biological tests were essential to the early diagnosis, treatment and prognosis of dogs with tumors.

## 1. Introduction

Canine mammary tumors (CMTs) are the most common neoplasms in female dogs, and approximately 50% are malignant [1]. For most dogs suspected of having CMTs, owners take the dogs to the veterinary hospital for examination when a visible protrusion is observed, at which point only surgical resection can be performed [2]. However, the high recurrence and metastasis rate of CMT results in poor prognosis, therefore, timely diagnosis is essential [3]. Diagnosis of CMTs in the early stage is one of the significant aspects of tumor treatment. Among various diagnosis platforms, X-ray, ultrasonography and histopathology can provide valuable data on CMTs. Moreover, serology and molecular biology can be employed as auxiliary diagnosis tools for CMTs, and blood detection can evaluate the systematic inflammation [4]. Several reports have demonstrated the applicability of imaging in the evaluation of mammary tumors in canines [5]. Certain data were recorded for each X-ray study, including abnormal shading (presence or absence), location of the mass (left, right, central), mass size, mass volume and the presence of metastases [6]. Ultrasonography is a non-invasive, well-tolerated technique that allows a real-time evaluation of mammary tissue. According to the literature, ultrasonography characteristics of healthy dogs are regular shape, circumscribed and regular margins and homogenous ecotecture, whereas the presence of irregular shape, ill-defined and non-circumscribed margins, homogenous ecotecture, anterior echogenic rim, acoustic posterior shadowing and vertical orientation may be suggestive of tumors [7]. As one of the common methods, histopathology is usually used to differentiate benign from malignant tumors [8]. Hematology, including complete blood count, blood chemistry profile and C-reactive protein (CRP) evaluation [9,10], is essential for early detection, timely diagnosis and treatment of diseases.

The human epidermal growth factor receptor 2 (HER-2) is a well-known negative prognostic factor in breast cancer. In veterinary oncology, the role of HER-2 has been investigated in CMTs by in situ hybridization and immunohistochemistry; however, investigations of serum using an enzyme-linked immunosorbent assay (ELISA) are limited [11]. E-cadherin and N-cadherin are cell adhesion molecules with pivotal roles in epithelial cell behavior, tissue formation, and suppression of cancers [12,13]. Vimentin is a major constituent of the intermediate filament family of proteins. In recent years, Vimentin has been recognized as a marker for epithelial-mesenchymal transition (EMT) and is overexpressed in canine tumors [14]. Tumor markers play an important role in the diagnosis, monitoring and prognosis of many tumors. The carcinoembryonic antigen (CEA), carbohydrate antigen 15-3 (CA15-3) and serum ferritin (SF) are typical tumor markers that have potential value in the diagnosis of mammary tumors [15,16]. In recent decades, many studies concerning biomarkers of the mammary glands have been conducted in both human and veterinary patients. However, no ideal biomarker has been found to date. An optimal biomarker should be easily measured in the blood, and its concentrations should only be elevated in cases of malignant tumors [17]. Therefore, this study was conducted to detect these tumor markers and cell adhesion molecules by serology and molecular biology for the preventive treatment of CMTs. Enzyme-linked immunosorbent assay (ELISA) can guide clinicians in diagnosing and monitoring diseases due to its high sensitivity in detecting very low-concentrations of molecules [18]. Quantitative PCR (qPCR) is one of the most common techniques for the quantification of nucleic acids in biological and environmental samples, and it has been the gold standard for gene expression analysis. Therefore, in this study, we identified CMTs via clinical observation, X-ray, ultrasonography and histopathology. We then analyzed the inflammatory status of dogs with CMTs through blood analysis, as complete blood count, blood chemistry profile and CRP. Moreover, several tumor markers and cell adhesion molecules in tumor tissues of dogs were determined by qPCR: HER-2, E-cadherin, N-cadherin, Vimentin, CEA, CA15-3 and SF. HER-2 was further tested in the sera of dogs with CMTs and healthy dogs. The results provide a reference for the prevention and treatment of CMTs in clinical veterinary medicine. In addition to clinical examination, biomarker tests have several advantages over histopathology, as the procedure of detection is non-invasive, and it can reveal dynamic changes in physiological and pathological states before clinical signs appear; therefore, it is used for the early detection of cancers. Early detection, diagnosis and treatment can improve survival and quality of life in dogs, which is significant for mammary tumor research. In the study, we have measured biomarkers in dogs by serological and molecular biological assay in the early diagnosis of tumors and assessment of disease progression and response to chemotherapy.

## 2. Materials and Methods

### 2.1. Animals and Sampling

Tumor, tumor-adjacent healthy tissues, blood and serum samples were collected from 30 mammary tumor-bearing dogs, and blood and serum samples were collected from 30 healthy dogs in Jilin Province, China, between 2018 and 2021. Canine tumor data were collected from several animal hospitals. The data included breed, age, sex, spayed status, geographical region, somatotype, number and location of the tumor. The tumor-adjacent healthy tissue was sampled about 1 cm from the tumor center. Tumor tissues and tumor-adjacent healthy tissues were placed into sterile 1.5 mL tubes after surgery to ensure hygienic sampling and were stored with serum samples at −80 °C refrigeration (ultralow temperature freezer) (TSX70086V, Thermo Scientific, Waltham, MA, USA). Blood samples were tested for routine blood and biochemistry within 4 hours of sampling.

### 2.2. Clinical Examinations

#### 2.2.1. Clinical Tumor Examinations

First, some clinical information about the dogs was collected, including the sex, age, breed, past spay/neuter and medical history. Subsequently, the chest and abdomen were observed for obvious lumps, regularity of shape, firmness to palpation, painful reactions and spillage when the nipples were squeezed. 

#### 2.2.2. Imaging Exams

The dogs were examined by X-ray (AVchoice 400 Plus, DEL MEDICAL, Bloomingdale, Naperville, IL, USA) and ultrasonography (MyLab™ Six VET, Esaote, Genoa, Italy) to confirm the presence or absence of tumor and organ metastases, such as the presence of abnormal shadows and cystic, anechoic areas, hyperechoic areas or acoustic shadowing on the other organs.

### 2.3. Pathology Examination—Hematoxylin and Eosin Staining

The dogs underwent excisional mastectomy. After the operation, a small section of tissue from the suspected region was removed, processed and stained with hematoxylin and eosin (H&E) (G1120, Solarbio, Beijing, China). Subsequently, the structure of the tissue from the glass slides was observed to differentiate them as benign or malignant. Histopathological images of the tumor were analyzed at different magnifications to assess the cellular and tissue-level variations. The following are the most significant criteria for the diagnosis of malignant mammary tumors in dogs based on H&E-stained sections: tumor type, significant nuclear and cellular pleomorphism, mitotic index, presence of randomly distributed areas of necrosis within the neoplasm, peritumoral and lymphatic invasion and regional lymph node metastasis. Furthermore, the degree of the malignant mammary tumors was graded according to a canine-adapted histological grading system, and according to the Tumor–Node–Metastasis (TNM) system for CMTs to determine the stage [19].

### 2.4. Hematology Examination

#### 2.4.1. Blood Routine Examination

Hematology provides valuable data for the nutritional status and clinical diagnosis of animals. Blood samples were collected from the dogs with CMTs and the healthy dogs, and a complete routine blood analysis was performed. For the routine analysis, 2 mL of blood was drawn into Ethylene Diamine Tetraacetic Acid (EDTA) (E8040, Solarbio) tubes. Complete blood counts were analyzed with a BC-5000 Vet Hematology Analyzer (Mindray, Shenzhen, China) within 4 h of collection. Common indicators such as lymphocytes, neutrophils and white blood cells were used to assess physical condition and to check for disease in the dogs.

#### 2.4.2. Blood Biochemical Examination

Accurate blood biochemical examination is essential for early detection and timely diagnosis and treatment of diseases. For biochemical analysis, 2 mL of blood was drawn into a tube without anticoagulant. Non-anticoagulative blood samples were centrifuged at 1200 rpm for 10 min to obtain serum. Serum biochemical analyses were performed using the SMT-120V (Seamaly, Chengdu, China). Indicators such as alkaline phosphatase, total bilirubin, total protein, albumin, globulin, alanine transaminase, γ-glutamyl transpeptidase, blood urea nitrogen, creatinine, glutamic acid and calcium were used to assess the physical condition and blood status of the dogs.

#### 2.4.3. C-Reactive Protein (CRP) Examination

CRP is an acute-phase plasma protein. It was discovered in 1930 and is well-characterized as an indicator of systemic inflammation, mostly produced in response to infection, tissue damage and stimulation of malignant tumors; it is mainly used in veterinary clinics to assess the severity of systemic inflammation in dogs and to monitor the effectiveness of treatment. For CRP analysis, 2 mL of blood was extracted into a tube without anticoagulant using the SMT-120V (Seamaly, Edmonton, AB, Canada). CRP was used to assess the inflammation in dogs.

### 2.5. Serological Molecular Biological Detection-ELISA

Using an ELISA kit (Canine EGFR2 ELISA KIT, RayBiotech, Atlanta, GA, USA), we detected HER-2 protein in the sera of dogs suffering from tumors and healthy dogs. First, serum samples from dogs with CMTs and healthy dogs and ELISA plates were placed at room temperature for 20 min. Then, 50 µL of the standard was added to the enzyme plate, and 40 µL of sample dilution was added to the sample wells, in addition to 10 µL of sample to be tested. Then, 50 µL of enzyme standard reagent was added to each well (except the blank wells) and the plate was incubated for 60 min at 37 °C. After discarding the liquid, each well was washed five times. Then, 50 µL of Color Developer A was added into each well, with an addition of 50 µL of Color Developer B, which was incubated at 37 °C for 15 min under dark conditions. Finally, 50 µL of termination solution was added to terminate the reaction (at which point the blue color turned yellow). The absorbance of each well at 450 nm was measured (Multiskan™ FC, Thermo Scientific, Waltham, MA, USA) and a standard curve graph was used to calculate the concentrations of the samples.

### 2.6. Molecular Biological Detection

#### 2.6.1. DNA Harvesting

Samples of tumors (100 mg) were homogenized with 1 mL of TRIzol solution (15596026, Thermo Fisher Scientific, Waltham, MA, USA). Total RNA was extracted according to the manufacturer’s instructions. The amount and purity of RNA in the sample was measured using a Nano Drop 1000 spectrophotometer (N anoPhotometer-NP80, Implen GmbH, München, Germany). The RNA sample absorbance ratio at A260:280 nm was approximately 2.0.

Each sample of total RNA (1 μg) was reverse transcribed to cDNA in a total of 20 µL using the HiScript^®^ II Q RT SuperMix for qPCR (+gDNA wiper) (Vazyme, Nanjing, China). The genome DNA removal reaction mixture consisted of 4 µL of 4× *g* DNA WIPER Mix, 1 µg of template DNA and RNase-free ddH_2_O to a total volume of 16 µL, and the reaction was performed for 2 min at 42 °C for 1 cycle. Then, 4 µL of 5× *g* HiScript Ⅱ qRT SuperMix Ⅱ Reaction Solution was added, and the reverse transcription reaction was performed for 15 min at 50 °C and 85 °C for 5 s followed by 1 cycle. The obtained cDNA was stored at −80 °C until use.

#### 2.6.2. Primer Design

The sequences of Vimentin, E-cadherin, N-cadherin, CA15-3, CEA, HER-2, SF and GAPDH were downloaded from GenBank, and aligned using the Clustal W algorithm of the MegAlign program in DNAstar 7.01 bioinformatics software (DNASTAR Inc., Madison, WI, USA). Eight pairs of specific primers were designed by Primer Premier 5.0 (PREMIER Biosoft International, Palo Alto, Santa Clara, CA, USA) based on the high conserved sequences of HER-2, E-cadherin, N-cadherin, Vimentin, CEA, CA15-3, SF and GAPDH (Table 1). All primers were synthesized by Jilin Kumi Biotechnology Co., Changchun, China.

#### 2.6.3. Conventional PCR Amplification

PCR amplification of HER-2, E-cadherin, N-cadherin, Vimentin, CEA, CA15-3, SF and reference genes was performed using the PCT-200 Peltier thermal cycler (MJ Research, Waltham, MA, USA). PCR was performed in a 25-μL reaction volume that contained 12.5 μL 2×Taq PCR Master Mix (P112-01, Vazyme), 3 μL cDNA template, 0.5 μL of each primer and 8.5 μL deionized water. The PCR parameters were 94 °C for 2 min; followed by 35 cycles of 94 °C 30 s, 58 °C 30 s and 72 °C 15 s, with a final extension at 72 °C for 10 min. Amplification products were visualized via 1.2% agarose gel electrophoresis (1645052, 1704486, Bio-Rad, Hercules, CA, USA).

#### 2.6.4. Real-Time PCR 

Using the Mx3005P qPCR System (Agilent Technologies, Santa Clara, CA, USA), we performed the real-time PCR amplification of HER-2, E-cadherin, N-cadherin, Vimentin, CEA, CA15-3 and SF. Real-time PCR was performed in 20-μL reaction volume, consisting of 10 μL FastStart Universal SYBR ^®^ Green Master (ROX), 1 μL cDNA template, 0.5 μL of each primer and 8 μL ddH_2_O. The real-time PCR parameters were 94 °C for 5 min, followed by 40 cycles of 94 °C 30 s, 55 °C 30 s, and 72 °C for 1 min, with a final extension of 72 °C for 10 min, 95 °C for 1 min, 55 °C 30 s and 95 °C 30 s. Ct was obtained by the Mx3000/Mx3005P real-time PCR system, and SD values were calculated with SD = STDEV (C1, C2, C3) to determine SD < 0.5 and the formula was calculated according to the 2^−∆∆Ct^ method as follows: ∆∆Ct = ∆Ct (experimental group) − ∆∆Ct (control group), ∆∆Ct (experimental group) = Ct (gene of experimental group) − Ct (gene of experimental group internal reference), ∆Ct (control group) = Ct (gene in the control group) − Ct (gene in the control inner reference group).

### 2.7. Statistical Analysis

Differences between groups were analyzed by GraphPad Prism 7.0(GraphPad Software, San Diego, CA, USA) software, and the experimental data were presented as mean ± standard deviation (SD). *p* < 0.05 was considered statistically significant.

## 3. Results

### 3.1. Clinical Examination

**Figure 1:**
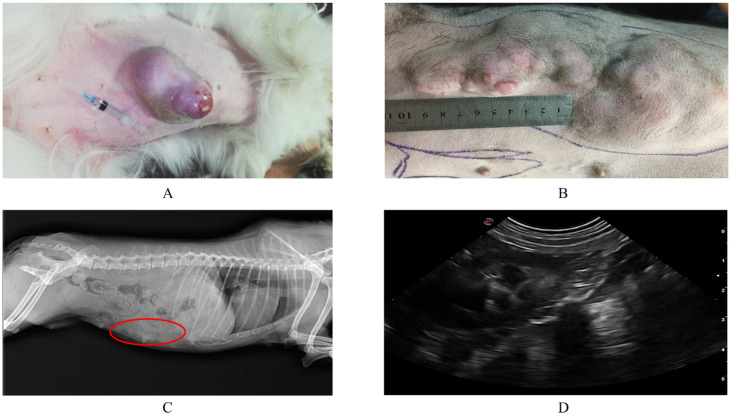
Clinical examination of canine mammary tumors. (**A**,**B**) are clinical observation pictures of canine mammary tumor diseases; the affected dogs were observed to have a distinct mass in the thorax, irregular in shape and sliding on palpation. Tumors were breakable, bleeding and ulceration during growth. (**C**) is a lateral view of the X-ray, the circle where a mass with slightly stronger density than the surrounding tissue is seen, soft tissue opacity structures were observed along the bilateral peripheral abdomen, with no obvious metastasis of the tumor. (**D**) is an ultrasonography examination with normal organ echo, and without abdominal metastases detected.

The mean age of the 30 dogs in this study was 8.9 years and ranged from 1 to 19 years. Poodles are the most represented in the study. The tumor size ranged from 0.2 cm to 2.9 cm (1.73 ± 0.56 cm, mean ± SD). Tumor localization was more common in the inguinal last two pairs of mammary glands, which was consistent with previous research [20]. Upon clinical examination, the dogs suffering from tumors had a distinct mass on the abdomen, in the thorax, irregular in shape and sliding on palpation (Figure 1A,B), and X-ray (Latero-lateral and ventro-dorsal views) revealed a mass-like effect with a slightly stronger density than the surrounding tissue in the mammary gland, without metastasis to the lungs (Figure 1C). Ultrasonography of the abdomen revealed normal organ echo, without metastases in the abdomen (Figure 1D).

### 3.2. Histological Examination

**Figure 2:**
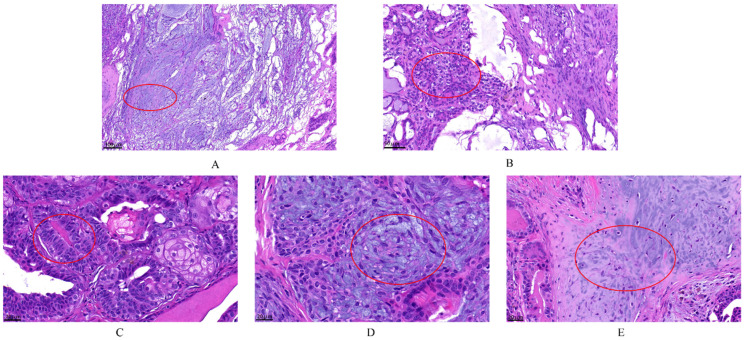
The histopathological examination of canine mammary tumors. The dye was Hematoxylin and Eosin (H&E) (G1120, Solarbio). (**A**,**B**) complex adenoma: There is pleomorphism of the epithelial cells that line the tubules but not the fusiform myoepithelial cells. Complex adenoma has both epithelial (tubular) and myoepithelial proliferation. This neoplasm consists of two cell populations and variable amounts of fibrous stroma. The cells are cuboidal to columnar and have a moderate amount of eosinophilic cytoplasm. Nuclei are round to oval with finely stippled or marginated chromatin and a single central basophilic nucleolus. Anisokaryosis and anisocytosis are usually minimal. The second population is composed of spindle to stellate cells with poorly demarcated cell borders and a moderate amount of cytoplasm. Nuclei are round to fusiform with finely stippled chromatin and a single nucleolus. (**C**–**E**) carcinoma arising in benign mixed tumor grade I: The malignant epithelial component forms tubules, and the mesenchymal component consists of islands of bone. The first population is composed of cuboidal cells arranged in irregular tubules (see (**C**) tumors break down during the growth). The cells have clear but irregular nucleoli and increased mitosis. The nucleolus is irregular. The second population is composed of spindle-shaped cells (myoepithelial cells; see (**D**)). These cells have a moderate amount of basophilic cytoplasm with poorly demarcated cell borders. The third component consists of foci of cartilage tissue, which exhibit no atypia (see (**E**)). There is an infiltrative growth pattern.

According to the histopathological classification of CMTs [8], histopathological sections of the tumors were used to diagnose the type of tumor. A total of 30 dogs were identified to suffer from CMTs, with 63.33% (19/30) malignant and 36.67% (11/30) benign. The benign tumors included six ductal adenomas, three complex adenomas and two benign mixed tumors, and the malignant tumors included eight mixed carcinomas, six tubular carcinomas, four ductal carcinomas, and one carcinoma arising in mixed mammary tumor. The malignant tumors were clinically staged according to the modified World Health Organization (WHO) clinical staging system [21] and were divided into the following categories: three mixed carcinomas grade I, three mixed carcinomas grade II, two mixed carcinomas grade III, three tubular carcinomas grade I, two tubular carcinomas grade II, one tubular carcinoma grade III, one ductal carcinoma grade I, one ductal carcinoma grade II, two ductal carcinomas grade III and one carcinoma arising in mixed mammary tumor grade I. Data on the clinical stage of the malignant tumors revealed stage I > stage II > stage III. The observed pathological changes are circle in red in Figure 2. In the benign mammary tumor (Figure 2A,B), epithelial (tubular) and myoepithelial proliferation was observed; this neoplasm consisted of two cell populations and variable amounts of fibrous stroma, and the nuclei were round to oval with finely stippled or marginated chromatin and a single central basophilic nucleolus. The malignant tumors (Figure 2C–E) had a malignant epithelial component and benign mesenchymal component, which were cartilage tissue. This neoplasm was characterized by the presence of three cell populations supported by fibrovascular stroma. Furthermore, there were numerous mitotic and infiltrative growth patterns, with significant pleomorphism of the epithelial component. 

### 3.3. Blood Analysis

**Figure 3:**
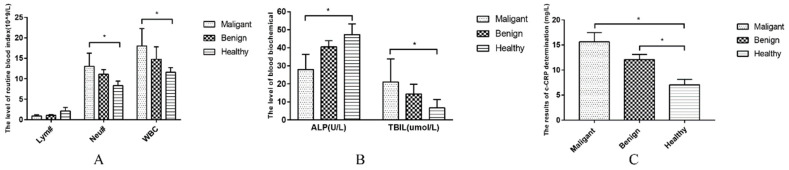
Comparison of blood examination results in each group. (**A**) Comparison of Lym#, Neu# and WBC levels in each group; (**B**) Comparison of ALP and TBIL levels in each group; (**C**) Comparison of CRP levels in each group. Note: Highly significant (*p* < 0.05) as indicated by *.

The blood counts of the dogs suffering from CMTs were analyzed in this study via routine blood analysis (Table 2). Dogs with malignant tumors had a lower lymphocyte (Lym) compared with healthy dogs (*p* < 0.05), with significantly higher levels of white blood cells (WBC) and neutrophils (Neu) (*p* < 0.05) (Figure 3A). Following blood biochemical examination, the serum biochemical values of dogs with benign tumors and healthy dogs were normal without a significant difference. (*p* > 0.05) (Table 3). A lower level of alkaline phosphatase (ALP) and higher total bilirubin (TBIL) were observed in dogs suffering from CMTs compared with healthy dogs (*p* < 0.05) (Figure 3B). Regarding CRP inflammatory tests, canine-CRP (c-CRP) was in the normal range in healthy dogs; however, it was significantly higher in dogs with tumors compared with healthy dogs (*p* < 0.05) (Table 4), which was also significantly different between dogs with malignant and benign tumors (Figure 3C).

### 3.4. Serological Molecular Biological Detection-ELISA

**Figure 4:**
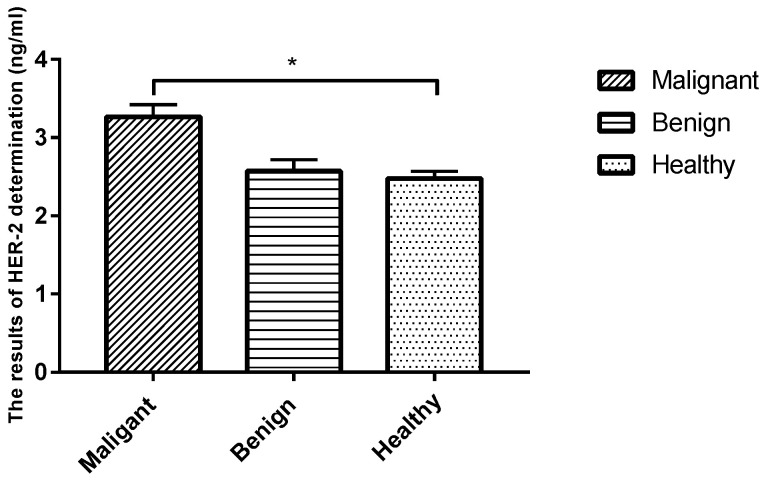
Comparison of serum HER-2 levels in each group. Note: Highly significant (*p* < 0.05) as indicated by *.

The sera of healthy dogs and dogs suffering from tumors were evaluated using a HER-2-ELISA kit, and the corresponding OD values were measured using a spectrophotometer at 450 nm. Analysis revealed that the level of tumor marker HER-2 in the serum was significantly higher in the malignant CMT group than in the benign group and the healthy control group (*p* < 0.05), while the expression level in the benign CMT group was not significantly different from that of the healthy control group (*p* > 0.05) (Table 5 and Figure 4).

### 3.5. Molecular Biological Detection-qPCR

**Figure 5:**
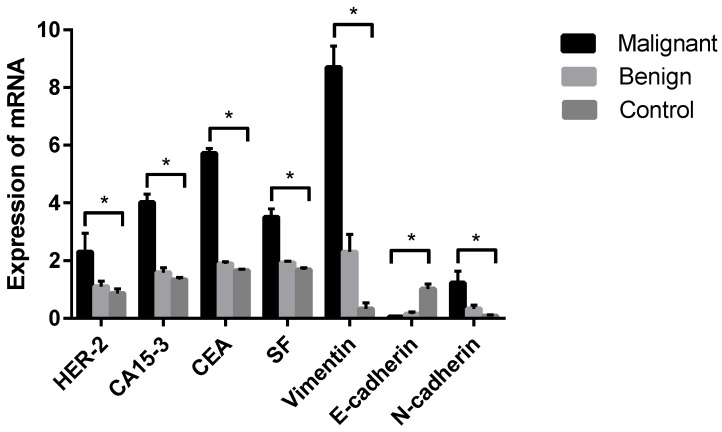
Comparison of HER-2, CA15-3, CEA, SF, Vimentin, E-cadherin and N-cadherin mRNA in each group. Note: Control means tumor-adjacent healthy tissues. Highly significant (*p* < 0.05) as indicated by *.

PCR products targeting HER-2, E-cadherin, N-cadherin, Vimentin, CEA, CA15-3, SF and GAPDH were all present in specific bands following visualization on 1.2% agarose gel electrophoresis (data not shown), suggesting that each pair of primers was specific to the target fragment. qPCR was further established in this study to analyze the differences in tumor markers between dogs with CMTs and healthy dogs. The results revealed that the relative expression levels of HER-2, CA15-3, CEA, SF, Vimentin and N-cadherin tumor marker mRNA were significantly higher in malignant CMTs than those in the benign and healthy groups (*p* < 0.05); however, there were no significant differences between the benign tumor group and the healthy control group (*p* > 0.05) (Table 6 and Figure 5). On the contrary, the expression level of the E-cadherin tumor marker mRNA in the healthy control group was significantly higher than in the benign tumor group and the malignant tumor group (*p* < 0.05), with no significant differences between the benign tumor group and malignant tumor group (*p* > 0.05).

## 4. Discussion

Mammary gland tumors are seen in female dogs of all ages and often lead to death due to their extreme aggression and high recurrence rate. Therefore, diagnosis is important for the prevention of progression and for CMT treatment. In this study, dogs with CMTs presented to the veterinarian with one or more nodules within the mammary gland. When tumors were present, the dogs showed non-specific symptoms such as fatigue, lethargy, weight loss, dyspnea, cough, lymphedema or lameness. The results of our study were in agreement with the results of a previous study [22]. Routine blood analysis revealed that monocyte and lymphocyte levels in dogs with CMTs were lower than in healthy dogs, but leukocyte levels were higher in dogs with CMTs. This observation suggests that the reduction in monocyte and lymphocyte levels and the increase in leukocytes may be related to the occurrence or development of CMTs. In blood biochemical examination, ALP and TBIL in dogs with CMTs were higher than in healthy dogs, and this difference may be caused by the tumors. Moreover, ALP was associated with prognosis, which further provides a basis for subsequent diagnosis and treatment. [23]. As the tumor grows, the level of CRP continues to rise; this is in line with previous studies [24]. This “acute-phase response”, in which CRP rapidly changes in inflammation and tissue injury in response to a variety of stimuli, was observed with the progression of some malignancies and in the activity of various diseases [25]. Changes in these indicators indicate multiple direct or paraneoplastic changes associated with tumors that can be detected/suspected by hematology examination. To our knowledge, these changes have not been mentioned in previous studies. Regarding serological molecular biological detection, the feasibility of using human antigen kits to determine HER-2 serum concentrations in dogs was demonstrated. The HER-2 serum concentrations were significantly different between healthy dogs and dogs with malignant tumors. However, these results are in disagreement with the results of a previous study [26]. ELISA for serum HER-2 is a dynamic test, however there is randomness in clinical samples that can lead to variation in the results. Previous studies have shown that HER-2 over-expression was detected in CMTs. Several studies have attempted to identify a similar prognostic role of HER-2 expression in canine mammary carcinomas. In previous investigations, HER-2 expression was found to be associated with some well-known morphological indicators of poor prognosis [11,27,28]. These were also demonstrated in our experiment. Moreover, ELISA for serum HER-2 can be performed at any time and can be used when primary tumor samples are unavailable, eliminating the need for a biopsy [29]. In this study, mRNA expression of HER-2, E-cadherin, N-cadherin, Vimentin, CEA, CA15-3 and SF was measured in CMT tissues by qPCR. The expression of these tumor markers (except for E-cadherin) in the malignant tumor group was significantly higher than that in the benign group and the healthy control group (*p* < 0.05), however, there was no significant difference between the benign mammary tumor disease group and the healthy control (*p* > 0.05). The mRNA expression of E-cadherin in tumor tissues was significantly lower than that in tumor-adjacent healthy tissues. This result was in line with some in-vitro studies, and a previous study demonstrated reduced expression of E-cadherin in malignant CMTs compared with normal mammary glands, suggesting that down-regulation of E-cadherin was a common event in canine mammary tumors [30]. Deregulation of E-cadherin is related to the infiltrative and metastatic ability of the tumor, due to of disruption of the E-cadherin, with consequent loss of cell adhesion and a concomitant increase in cell motility. Moreover, a reduction in E-cadherin expression was significantly associated with infiltrative growth and vessel invasion [31]. In conclusion, there is a significant relationship between loss of E-cadherin expression and other known factors for poor prognosis in CMTs, such as tumor size, ulceration, histological type, type of growth, lymph node metastasis and necrosis [31,32,33]. This indicates that a loss of E-cadherin expression may have prognostic value in malignant CMTs. The expression level of E-cadherin in tumor tissue is correlated with disease progression, and it can be used as a diagnostic marker for mammary tumors [34]. Further evidence should be sought in future studies in which the clinical course of the disease is carefully monitored.

From a clinical point of view, imaging was used to further assess for the presence of tumors that are not visible to the naked eye, and to provide complete imaging information [22]. Pathology examinations are mainly used to diagnose mammary tumors. The evaluation of the morphological appearance and malignant growth behavior of the tumor help to identify the nature of the tumor (benign or malignant) [4]. However, pathological examinations have limited predictive value for the prognostic status of patients, and X-ray examinations also have limitations. For example, overlapping images cannot always be distinguished. Hematological examinations cannot directly determine the presence of tumors, but they can be used as auxiliary tests to guide further diagnosis. Therefore, we discussed the importance of serological tests and molecular biological tests in the diagnosis of CMT. To date, the most studied and reliable biomarkers of CMT are HER-2, E-cadherin, N-cadherin, Vimentin, CEA, CA15-3 and SF, which can be detected in both serum and tissue samples using different molecular methods. Notably, these biomarkers can be used for the early detection and prognosis of CMT. Early detection, diagnosis and treatment can improve survival and quality of life in both humans and dogs, which is significant for mammary tumor research [35,36]. In both human and veterinary medicine, many studies on the biomarkers of mammary gland tumors have been conducted. In recent years, the use of serological molecular biological detection and molecular biological detection to assess the status and prognosis of tumor patients has shown several prospective clinical applications [4], and these methods are important in assessing whether a mammary tumor will recur after surgery [37]. In addition, these methods can be used to monitor tumors through changes in blood composition and to detect the presence of tumors much earlier than conventional diagnostic methods. Serological tests and molecular biological tests have advantages over histopathology, as the procedure of detection is non-invasive, and they can reveal dynamic changes of physiological and pathological states before clinical signs appear. Furthermore, serological and molecular biological testing is more cost-effective than traditional diagnostic methods [38,39]. It is not only suitable for early diagnosis of CMTs, but also for occasional physical examination of healthy dogs. However, the early detection of CMTs, the causative factors and the clinical evaluation of tumors will continue to be a challenge. For this reason, the early detection of CMTs is crucial for the dog’s clinical outcomes. Unfortunately, serological canine tumor biomarkers are rarely applied in veterinary clinics. To obtain more reliable results, we always recommend the evaluation of more than one biomarker [40]. Measurement of biomarkers in dogs by serological and molecular biological assays is a milestone in the early diagnosis of tumors and assessment of disease progression.

## 5. Conclusions

In our study, CMTs were diagnosed by clinical detection and histopathology. In the hematology, there were significant changes in inflammatory markers. In the molecular biology, the expression levels of HER-2, E-cadherin, N-cadherin, Vimentin, CEA, CA15-3 and SF were significantly different between dogs with tumors and healthy dogs. The measurement of biomarkers in dogs by serological and molecular biological assays represents a milestone in the early diagnosis of tumors and assessment of disease progression and response to chemotherapy. Early detection, diagnosis and treatment can improve survival and quality of life in both humans and dogs, which is significant for mammary tumor research and is important when assessing whether a mammary tumor will recur after surgery. These findings need to be validated in future investigations, and tumor marker assays will soon be widely used in veterinary clinics if they are found to be reproducible.

## Figures and Tables

**Table 1:** Primers used for amplification of target genes in this study.

Name of Primer	Sequence (5′–3′)	Expected Product/Bp	Application
Vimentin-F	TACGCCAGCAATATGAAAGCG	112	Vimentin
Vimentin-R	AGGGCATCATTGTTCCGGTTA		
E-cadherin-F	TCCTGGGCAGGGTGAGTT	134	E-cadherin
E-cadherin-R	GAGGCCGCTTGACTGTAATC		
N-cadherin-F	AGCACCCTCCTCAGTCAACG	126	N-cadherin
N-cadherin-R	TGTCAACATGGTCCCAGCA		
CA15-3-F	CTGCTGGTGCTGGTCTGTGTTCTG	264	CA15-3
CA15-3-R	GGCTGCTGGGTTCGGGTTCAT		
CEA-F	GCCAGATTCTAACGCTCACGGATAG	165	CEA
CEA-R	AATCATCTTCCACATCCAGCCTTACAG		
HER-2-F	AAGTGCTGGATGATAGACTCTGAATGC	157	HER-2
HER-2-R	GTAGTGAACGGTAGAAGGTGCTGTC		
SF-F	GATGCTGCTTCTGGTATGTCCTATCTC	146	SF
SF-R	GAATACACTCCACCATCCTCTTGACG		
GAPDH-F	ATGGTGAAGGTCGGAGTGAA	163	GAPDH
GAPDH-R	GGAATTTGCCGTGGGTAGAAT		

**Table 2:** Results of routine blood tests.

Parameters	Min–Max	Unit	Reference Range	Value
White blood cells (WBC)	9.59–26.73	10^9^/L	6.00–17.00	16.32
Neutrophil (Neu#)	6.79–18.84	10^9^/L	3.62–12.30	11.45
Lymphocyte (Lym#)	0.43–3.91	10^9^/L	0.83–4.91	44.31
Monocyte (Mon#)	0.13–2.66	10^9^/L	0.14–1.97	1.75
Eosinophil (Eos#)	0.05–1.50	10^9^/L	0.04–1.62	1.23
Basophil (Bas#)	0.00–0.08	10^9^/L	0.00–0.12	0.04
Red blood cells (RBC)	4.65–7.31	10^12^/L	5.10–8.50	7.21
Hemoglobin (HGB)	125–183	g/L	110–190	165
Red blood cell specific volume (HCT)	39.9–56.5	%	33.0–56.0	51.3
Mean corpuscular volume (MCV)	62.4–70.8	fL	60.0–76.0	65.2
Mean corpuscular hemoglobin (MCH)	22.3–27.8	pg	20.0–27.0	25.9
Mean corpuscular hemoglobin concentration (MCHC)	315–368	g/L	300–380	345
Red blood cell distribution width (RDW-CV)	13.2–16.1	%	12.5–17.2	15.2
Red blood cell distribution width-standard deviation (RDW-SD)	33.6–47.2	fL	33.2–46.3	45.3
Platelet count (PLT)	98–482	10^9^/L	117–490	467
Mean platelet volume (MPV)	8.4–12.9	fL	8.0–14.1	11.6
Platelet distribution width (PDW)	12.7–16.8		12.0–17.5	15.7
Plateletcrit (PCT)	0.12–0.53	%	0.090–0.580	0.49

**Table 3:** Results of Blood Biochemical Examination.

Parameters	Min–Max	Unit	Reference Range	Value
Total Protein (TP)	54–78	g/L	52–82	71
Albumin (ALB)	25–35	g/L	22–44	29
Globulin (GLO)	33.8–50.6	g/L	23–52	47.5
Alkaline phosphatase (ALP)	12–55	U/L	40–300	41
Total bilirubin (TBIL)	2.22–51.29	umol/L	2–15	39.89
Alanine transaminase (ALT)	16–68	U/L	10–118	51
γ-glutamyl transpeptidase (GGT)	1.25–1.83	U/L	0–7	1.46
Blood Urea Nitrogen (BUN)	2.9–5.9	mmol/L	2.5–9.6	3.4
Creatinine (CRE)	38–82	umol/L	27–124	74
Glutamic acid (GLU)	4.35–7.22	mmol/L	3.89–7.95	6.32
Calcium (Ca)	2.12–2.86	mmol/L	1.98–2.95	2.64

**Table 4:** Comparison of serum c-CRP levels between the different groups.

Grouping	c-CRP (mg/L)
Healthy	5.858 ± 2.609 *
Benign	9.883 ± 2.805
Malignant	14.58 ± 2.516

Note: The difference between the mammary tumor group and the healthy control group is highly significant (*p* < 0.05) as indicated by *.

**Table 5:** Comparison of serum HER-2 levels between the different groups.

Grouping	HER-2 (ng/mL)
Healthy	2.48 ± 0.09005
Benign	2.573 ± 0.1445
Malignant	3.264 ± 0.1591 *

Note: The difference between the mammary tumor group and the healthy control group is highly significant (*p* < 0.05) as indicated by *.

**Table 6:** The relative expression of HER-2, CA15-3, CEA, SF, Vimentin, E-cadherin and N-cadherin mRNA.

Grouping	HER-2	CA15-3	CEA	SF	Vimentin	E-Cadherin	N-Cadherin
Healthy	0.864 ± 0.1603 *	1.348 ± 0.0639 *	1.655 ± 0.0451 *	1.686 ± 0.0639 *	0.327 ± 0.2146 *	1.018 ± 0.1711 *	0.0862 ± 0.0314 *
Benign	1.104 ± 0.1871	1.587 ± 0.1667	1.898 ± 0.06784	1.929 ± 0.04963	2.307 ± 0.6025	0.157 ± 0.0661	0.3268 ± 0.1263
Malignant	2.307 ± 0.6374	4.024 ± 0.2801	5.728 ± 0.1618	3.511 ± 0.2836	8.706 ± 0.7283	0.052 ± 0.0267	1.226 ± 0.4087

Note: The difference between the mammary tumor group and the healthy control group is highly significant (*p* < 0.05) as indicated by *.

## Data Availability

Not applicable.
